# Prophylaxis of Grippe

**Published:** 1919-01

**Authors:** 


**_On the Prophylaxis of Grippe._** By CHAUFFARD, NETTER, VINCENT, ACHARD and BEZANÇON. *Bulletin de l'Académie de Médecine*, October 15, 1918.

MM. Chauffard, Netter, Vincent, Achard and Bezançon were charged by the Academy of Medicine with the drawing of a report on the prophylaxis of grippe.

The extreme contagiosity of the present epidemic recalls that of 1889. A very short contact with an infected person is enough to pass the disease, incubation is markedly short, sometimes only a few hours, and diffusion increases with promiscuity. This is why crowding in camps, theaters, cinemas and large shops has greatly contributed to the incidence of the disease.

A first attack does not immunize against a second one, but the greatest danger is that the grippe is an open door to very serious secondary infections. Some are pulmonary, some have the character of a general infection, such as in extensive septicemias, and some are intestinal.

The notion of the subdivisions of gripppe has shown that not only is it necessary to separate grippe patients from all others, but also to segregate them according to the subdivisions above mentioned.

In the private home, precautions should be taken exactly as they are in hospital wards. A special room must be given to the patient, strictly forbidden to all, save the attendant, who will wear a gown on entering the room.

The Home Secretary has ordered doctors to report the disease and has begged that isolation and disinfection should be carried out just as if public notification were compulsory.

Grippe being transmitted chiefly through the upper respiratory organs, the individual prophylaxis is to protect and disinfect carefully the nose and the mouth. One of the simplest and best ways is the use of a mouth wash with hot salt solution to which is added a small spoon of Labarraque liquor.

Whether in the home or in the hospital wards, attendants must wash their hands before each meal; the wearing of a mask is strongly urged, and should be made compulsory. In the hospitals there must be immediate widening of the spaces between the beds and removal of surplus patients, such as tubercular patients, to proper sanatoria.

Personal isolation, though rarely possible, stops the transmission of contagion. Even a muslin hanging from a string separating the beds will suffice to protect neighboring patients from the droplets of an infected one.

Concerning the general protective measures, an appeal was made not to frequent crowded halls such as theaters, cinemas and concert halls.

Where avoidance is impossible, such as in railways and subways, disinfection on a large scale should be attempted.

In crowded buildings there should be free, abundant aeration, and strict prevention of sweeping without a wet mop, since dry-sweeping disseminates dust and germs. The closing of schools where there have been cases of grippe is a necessary measure, and the necessity of complete disinfection of the premises is too obvious to be recommended. In all cases the floors should be washed thoroughly and repeatedly. Visitors should not be allowed near the patients.

Men gassed at the front are particularly susceptible to infections of the respiratory tract, and they ought to be warned to avoid contact with persons who have the grippe.

